# Changes in Heart Rate Variability During Heartfulness Meditation: A Power Spectral Analysis Including the Residual Spectrum

**DOI:** 10.3389/fcvm.2019.00062

**Published:** 2019-05-14

**Authors:** Anne Léonard, Serge Clément, Cheng-Deng Kuo, Mario Manto

**Affiliations:** ^1^HELB Ilya Prigogine, Brussels, Belgium; ^2^Division of Chest Medicine, Department of Internal Medicine, Changhua Christian Hospital, Changhua City, Taiwan; ^3^Department of Medical Research, Taipei Veterans General Hospital, Taipei, Taiwan; ^4^Service de Neurologie, ULB CHU-Charleroi, Charleroi, Belgium; ^5^Service des Neurosciences, University of Mons, Mons, Belgium

**Keywords:** meditation, heartfulness, heart rate variability, power spectrum, residual spectrum, vagal, sympathetic, paced breathing

## Abstract

**Background:** Meditation refers to a group of practices commonly proposed to treat stress-related conditions and improve overall wellness. In particular, meditation might exert beneficial actions on heart rate variability (HRV) by acting on autonomic tone with an increase in the vagal activity. The effects of heartfulness meditation (HM) on HRV remain poorly defined.

**Methods:** We investigated the effects of HM on HRV in a group of 26 healthy subjects. Subjects were regularly practicing this form of meditation on a daily basis. We assessed the HRV and residual HRV (rHRV) at rest and during meditation. We also used as control a period of respiratory rhythm imposed by an auditory signal, with the imposed breathing rhythm being identical to the spontaneous rhythm recorded during meditation.

**Results:** During deep meditation period, the standard deviation of RR intervals (SD_RR_), coefficient of variation of RR intervals (CV_RR_), and total power (TP) were decreased while the low-frequency power (LFP), normalized LFP (nLFP), and normalized residual LFP (nrLFP) were increased as compared with those at rest, suggesting that the global vagal modulation was suppressed while the baroreflex was increased during deep medication. At the end of meditation, the LFP, residual LFP (rLFP), nLFP, nrLFP, low-/high-frequency power ratio (LHR), and residual LHR (rLHR) were increased while the residual very low-frequency power (rVLFP), normalized high-frequency power (nHFP), and normalized residual HFP (nrHFP) were decreased, as compared with those during paced breathing, suggesting that the vagal modulation was decreased while the sympathetic modulation was increased by deep meditation. During paced breathing period, the SD_RR_, CV_RR_, TP, LFP, rLFP, nLFP, nrLFP, LHR, and rLHR were decreased while nHFP and nrHFP were increased as compared with at rest, suggesting that paced breathing could suppress the sympathetic modulation and enhance the vagal modulation.

**Conclusion:** HM can induce a suppression of global vagal modulation and increased the sympathetic modulation and baroreflex. In addition, paced breathing can suppress the sympathetic modulation and enhance the vagal modulation. Unlike studies using other types of meditation, we did not identify evidence of increased vagal tone during HM.

## Introduction

Heart rate variability (HRV) designates the continuous oscillations of successive RR intervals around the mean value (1, 2). The assessment of HRV by power spectral analysis allows extraction of the sympathetic and parasympathetic (vagal) modulations of heart rate (HR). Indeed, HRV results from the dynamic balance generated by the coactivation, co-inhibition, or reciprocal activation/inhibition of both the sympathetic and parasympathetic nervous systems (3). HRV is thus an indicator of regulation of the autonomous nervous system. This is particularly relevant in medicine since prolonged autonomic imbalance has been associated with a variety of somatic and mental disorders (4).

The terminology of meditation refers to a group of mental practices aiming to improve the overall wellness and to reduce stress. Meditation would be beneficial on a wide range of disorders (such as anxiety, depression, pain) and would exert positive effects upon HRV by increasing vagal tone. HRV was shown to be modulated during meditation and yoga practice (5, 6) with a positive effect on sympathovagal balance. Heartfulness meditation (HM) is a heart-based meditation practice aiming to reach a balance of mind (7). Participants redirect their mind toward the heart. Favorable effects on burnout and emotional wellness have been reported recently (7).

Although HM is used on a regular basis in many countries, its effects upon HRV are unknown. Our two goals were: (1) to evaluate whether HM increases HRV and residual HRV (rHRV), (2) to assess the effects of the respiration rhythm (spontaneously adopted by meditators during HM) upon HRV and rHRV, given the critical importance of respiratory patterns upon HRV spectra (8).

## Methods

The study was approved by the Ethical Committee of ULB Erasme (Reference 2016-521). All participating subjects signed a written informed consent after full explanation of the experimental procedures and before the experiment.

### Study Subjects

We investigated the effects of HM on HRV and rHRV in a group of 26 healthy subjects (17F/9M; mean age ± SD: 50.5 ± 8.6 years). Subjects were regularly practicing this form of meditation on a daily basis (mean duration of practice ± SD: 13.1 ± 7.4 years). A total of 30 subjects were included in the study. Data were not analyzable in 4 subjects due to ECG recording artifacts. The results shown correspond to *n* = 26 subjects.

### Inclusion and Exclusion Criteria

Each participant used to practice HM for 1-h on a daily basis for a minimum of 4 years. All subjects were no smokers and not suffering from chronic alcoholism. They had no history of respiratory, cardiac and neurologic diseases such as stroke, brain tumor, cerebral malformation, epilepsy, neurosurgical intervention, migraine, cranial trauma, depression, Parkinson disease, tone disorders. None of them had an implantable pacemaker. They were taking no psychotropic drugs or narcotic substances.

Participants were asked not to consume alcohol within 12 h, coffee/tea within 3 h, and could not practice sports during the 12 h before the experiment.

### Questionnaires

The following questionnaires were used:

- Freiburg Mindfulness Inventory (FMI): the French version has been used before the experimentation to measure dispositional mindfulness in daily activities. The score is related with years of experience.
- Mindfulness Attention Awareness Scale (MAAS)
  Attention is a common characteristic of different types of meditation and seems to improve as meditators gain experience. Each subject was requested to fill in the questionnaire before the experimentation with aim of measuring the attention through subjective experiences.
- Meditation Depth Questionnaire (MEDEQ)

Following the 30 min-meditation ECG recording, a self-report to measure the depth of the meditative state was submitted to each subject. Adapted from the MEDEQ by Ott in 2001 and Thomas and Cohen (9), this questionnaire consists of a visual analog scale rated from 0 (no meditation) to 10 (deepest meditation state). We added 4 time marks in order to have the same reference on the ECG for the 30-min-meditation state and to compare the self-report data for each meditator and the ECG recording.

### Monitoring of Heart Rate and Respiration

A non-invasive monitoring was performed using IWorx 214-Data Recorder (iworx, Dover, USA). The device includes a duo channel bio potential amplifier. Wires are connected to collect ECG peripheral data using five electrodes positioned on wrists and ankles according to the recommendations of the Association for the Advancement of Medical Instrumentation. Another cable is connected to a respiration monitor RM-204 employing piezo technology to follow the relative depth and frequency of breathing. The measuring element is mounted in a belt that straps around the chest of the subject. Labscribe 2 software was used to collect and analyze data from ECG leads I and II and breathing.

### HRV Parameters

HRV was computed from R-R intervals, as detailed previously (1, 2). The adjacent RR intervals were assessed for each study participant during 4 periods: during the rest period, during meditation (in the depth of meditation, at the end) and during the imposed respiratory rhythm period.

We assessed the HRV:

- (a) at rest
- (b1) during deep meditation
- (b2) at the end of meditation
- (c) during “paced breathing”: a control period with a respiratory rhythm imposed by an auditory signal. The imposed breathing rhythm was identical to the spontaneous rhythm recorded during meditation.

The area under the curve of the spectral peaks within the frequencies range of 0.01–0.4, 0.01–0.04, 0.04–0.15, and 0.15–0.40 Hz were defined as the total power (TP), very low-frequency power (VLFP), low-frequency power (LFP), and high-frequency power (HFP), respectively.

In order to normalize VLFP, LFP, and HFP, we used the power within the frequency range of 0.01–0.4 Hz (1, 2). The normalized VLFP (nVLFP = VLFP/TP) is an index of vagal withdrawal, renin–angiotensin modulation, and thermoregulation (10). The normalized low-frequency power (nLFP = LFP/TP) represents an index of combined sympathetic and vagal modulation (11) and an index of baroreflex (12, 13), and the normalized HFP (nHFP = HFP/TP) corresponds to an index of vagal modulation. The low-/high-frequency power ratio (LHR = LFP/HFP) is the index of sympathovagal balance.

### Respiratory Rate

The respiratory rate was determined in breaths/min (bpm).

### Residual HRV

The power spectrum of HRV was decomposed into a power-law function and a residual part of HRV (1, 2),

$$\begin{array}{l} \text{PSD}={{\text{F}}_{\text{rg}}\cdot \text{rPSD}=10}^{Y}\cdot \text{Fr}{\text{q}}^{\text{s}}\cdot \text{rPSD}, \end{array}$$

where the PSD is the traditional power spectral density, *F*_*rg*_ is the function of linear regression between log(PSD) and log(Frq) with slope “s” and Y-intercept “Y” within the frequency range from >0 Hz to the Nyquist frequency, “rg” stands for regression, Frq is the frequency, and rPSD is the residual PSD. The *F*_*rg*_ is a power-law function of Frq with exponent “s” and normalization constant 10^*Y*^.

Similar to the definition of traditional HRV measures, the area-under-the-curve of the spectral peaks within the range of 0.01–0.4, 0.01–0.04, 0.04–0.15, and 0.15–0.40 Hz in the residual power spectrum were defined as the residual total power (rTP), residual very low-frequency power (rVLFP), residual low-frequency power (rLFP), and residual high-frequency power (rHFP), respectively. The normalized rVLFP (nrVLFP = rVLFP/rTP), normalized rLFP (nrLFP = rLFP/rTP), normalized rHFP (nrHFP = rHFP/rTP), and residual low-/residual high-frequency power ratio (rLHR = rLFP/rHFP) were defined in similar ways to those of traditional HRV measures.

### Statistical Procedures

Statistical analysis was performed using Matlab and Sigmaplot^®^ (Jandel Scientific, Germany). Descriptive statistics were computed. Mean, median, quartiles, and SD values were extracted. Statistical significance was set at 0.05.

Data normality was assessed using the Shapiro-Wilk test. To test the difference in ECG parameters between conditions, a non-parametric analysis of variance on ranks was made using a Friedman test. When Friedman test was positive, *post-hoc* analysis was done with a Wilcoxon rank test. Comparisons of respiratory rhythm at rest and during meditation periods were done with Student *t*-test.

## Results

### Effects of HM on HRV and rHRV

**Figure 1:**
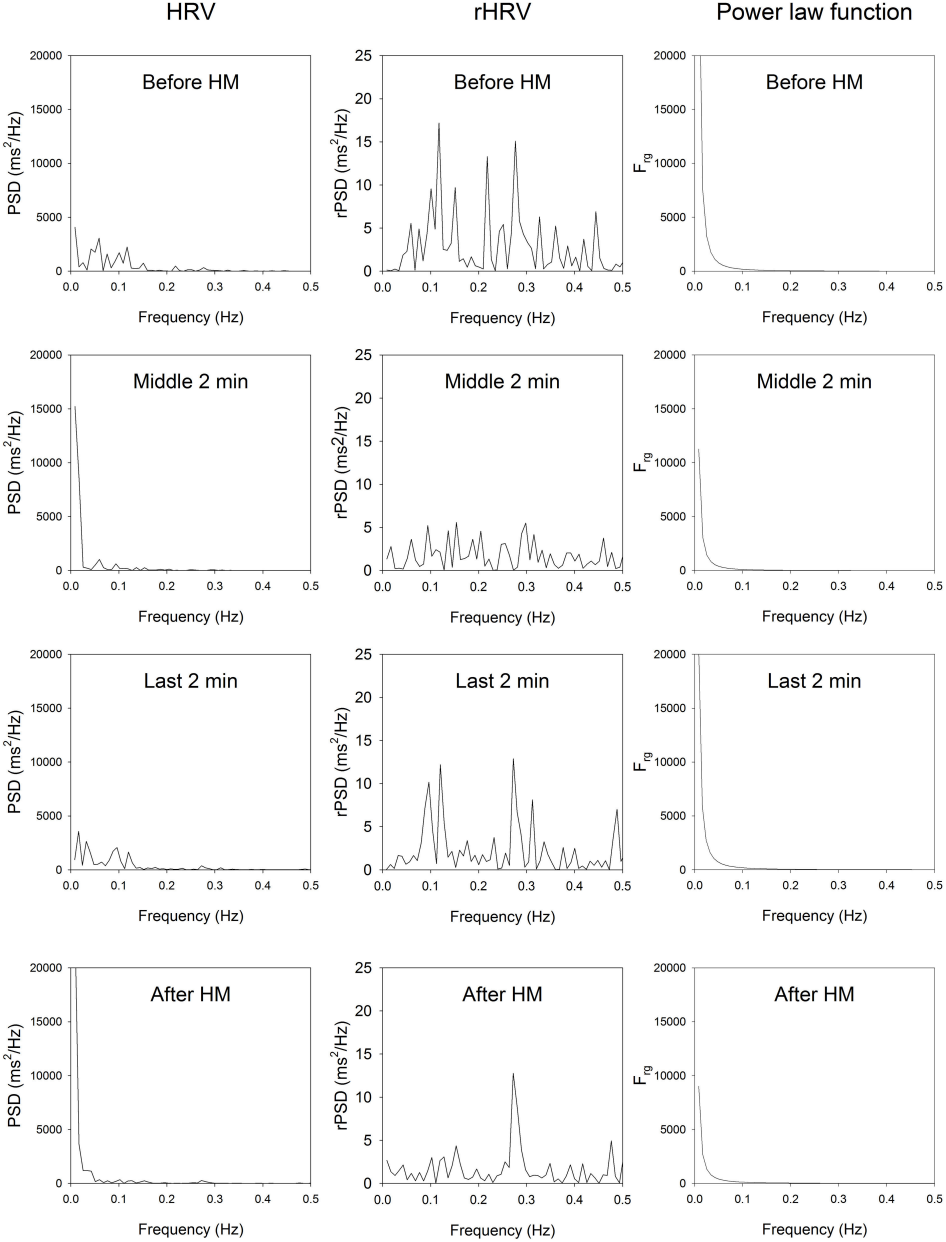
HRV spectrum, residual HRV spectrum and the power law function of a representative participant. Top, before HM; middle upper panels, middle of HM; middle lower panels, last 2 min of HM; bottom panels, imposed breathing after HM. In all stage of HM and after HM, the very low-frequency part of the rHRV is reduced, the low-frequency part of rHRV is reduced, while the high-frequency part of the rHRV is enhanced.

A typical example of the effects of HM upon spectra of HRV for one subject is illustrated in Figure 1. HM caused a reduction of standard deviation of RR intervals (SD_RR_), coefficient of variation of RR intervals (CV_RR_), and total power (TP).

Table 1 shows that during deep meditation period b1, the SD_RR_, CV_RR_, and TP were significantly decreased as compared with those measures at rest a, and the LFP, nLFP, and nrLFP were significantly increased as compared with those measures during the control period c when the breathing rhythm was paced in accordance with the spontaneous rhythm. At the end of meditation b2, the LFP, rLFP, nLFP, nrLFP, LHR, and rLHR were increased, while the rVLFP, nHFP, and nrHFP were decreased, as compared to the corresponding HRV and rHRV measures in the control period of paced breathing c. During the control period of paced breathing c, the SD_RR_, CV_RR_, TP, LFP, rLFP, nLFP, nrLFP, LHR, and rLHR were decreased, while the nHFP and nrHFP were increased, as compared with the corresponding HRV and rHRV measures at rest a.

**Table 1:** Comparison of HRV and rHRV measures among at rest (a), during deep medication (b1), at the end of medication (b2), and during the control period (c).

	**At rest (a)**	**Deep meditation (b1)**	**End of meditation (b2)**	**Control period (c)**	***P***
mRRI	842 (754–916)	833 (759–925)	815 (767–883)	822 (760–899)	0.361
HR	71.3 (65.5–79.5)	72.1 (64.9–79.0)	73.6 (68.0–78.2)	73.0 (66.7–78.9)	0.351
SD_RR_	28.1 (21.3–42.9)^#^	22.8 (20.4–38.2)^*^	31.0 (18.9–40.2)	24.0 (19.5–32.5)^*^	0.014
CV_RR_	0.033 (0.029–0.047)^#^	0.031 (0.026–0.042)^*^	0.036 (0.02–0.047)	0.027 (0.024–0.034)^*^	0.002
RMSSD	20.2 (11.6–32.7)	19.2 (12.0–33.4)	18.6 (12.2–28.3)	18.3 (12.3–27.4)	0.302
TP	390.2 (223.1–885.9)^#^	258.0 (194.8–712.6)^*^	467.4 (174.3–793.8)	279.0 (180.2–511.7)^*^	0.010
rTP	1.224 (0.935–1.689)	1.360 (1.083–1.641)	1.313 (0.982–1.652)	1.417 (1.115–2.387)	0.183
VLFP	82.2 (56.2–133.0)	67.6 (28.4–112.9)	64.2 (29.5–77.0)	51.4 (23.0–93.7)	0.059
rVLFP	0.028 (0.016–0.045)	0.033 (0.016–0.052)	0.020 (0.011–0.032)^#^	0.047 (0.031–0.067)	<0.001
LFP	144.1 (77.7–317.5)^#^	114.0 (45.5–180.6)^#^	134.0 (61.7–347.7)^#^	40.4 (25.3–82.8)^*^	<0.001
rLFP	0.375 (0.204–0.538)^#^	0.252 (0.188–0.479)	0.412 (0.260–0.689)^#^	0.187 (0.154–0.259)^*^	<0.001
HFP	83.7 (31.0–238.0)	83.7 (45.7–244.3)	60.8 (31.3–137.6)	74.2 (29.9–199.8)	0.477
rHFP	0.639 (0.424–1.110)	0.818 (0.585–1.261)	0.538 (0.430–1.207)	1.131 (0.665–1.778)	0.057
nVLFP	17.4 (13.0–28.2)	17.7 (11.6–30.2)	13.4 (10.3–18.3)	18.8 (9.8–28.2)	0.270
nrVLFP	1.86 (1.08–5.36)	2.21 (0.99–4.80)	1.54 (0.74–3.43)	3.18 (1.09–5.22)	0.073
nLFP	38.0 (18.4–3.6)^#^	35.1 (20.1–3.9)^#^	44.7 (20.6–4.0)^#^	21.0 (15.5–3.0)^*^	<0.001
nrLFP	33.11 (22.47–45.28)^#^	23.06 (16.74–38.53)^#^	37.75 (21.51–55.79)^#^	13.59 (9.30–25.48)^*^	<0.001
nHFP	24.6 (17.0–3.3)^#^	29.6 (19.2–3.8)	25.4 (0.2–4.0)^#^	34.7 (24.6–4.8)^*^	0.017
nrHFP	62.91 (52.35–76.83)^#^	73.66 (59.99–78.78)	58.26 (41.65–77.21)^#^	83.19 (65.61–88.81)^*^	<0.001
LHR	1.87 (0.72–3.99)^#^	1.16 (0.63–2.03)	2.56 (0.85–7.39)^#^	0.61 (0.29–1.92)^*^	<0.001
rLHR	0.52 (0.29–0.89)^#^	0.31 (0.21–0.64)	0.65 (0.28–1.38)^#^	0.17 (0.11–0.40)^*^	<0.001

* *P < 0.05 vs. at rest (a), ^#^P < 0.05 vs. during the control period of paced breathing (c)*.

### Effects of HM on Respiratory Parameters

**Figure 2:**
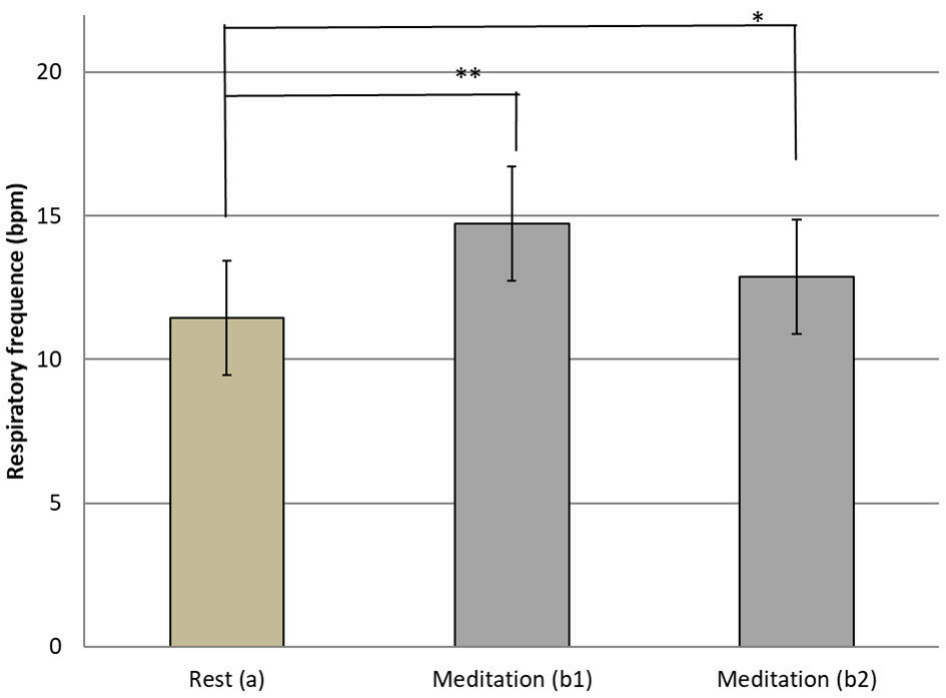
Means and SD Respiratory rythm at rest (a) and during meditation (b1) (b2). i*p = 0.02; **p < 10^−5^.

**Figure 3:**
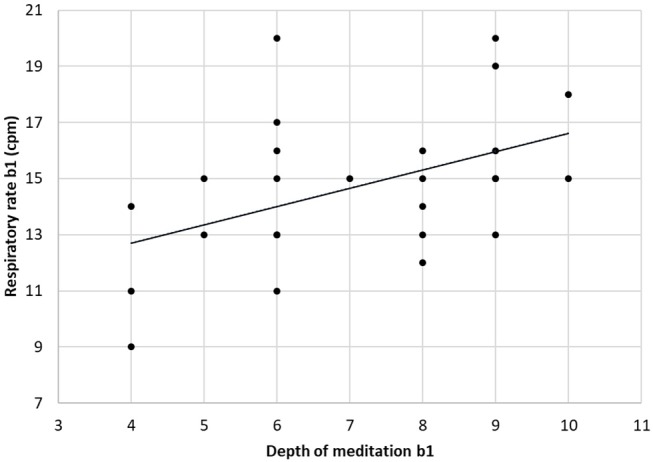
Linear correlation between respiratory rate and depth of mediation (ρ = 0.9685, *p* = 0.02).

**Figure 4:**
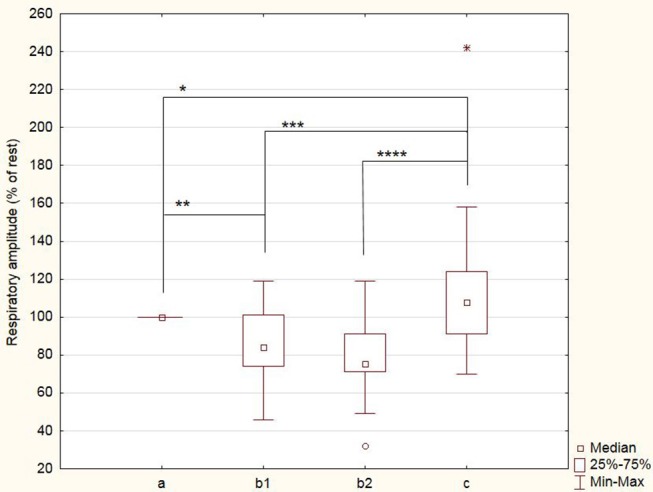
Breathing amplitude during meditation (b1) and (b2) and during control period of paced breathing (c). Amplitude are expressed as percent of rest condition. ^*^p = 0.04; ^**^p = 0.0006; ^***^p = 0.0005; ^****^p = 0.0002.

**Figure 5:**
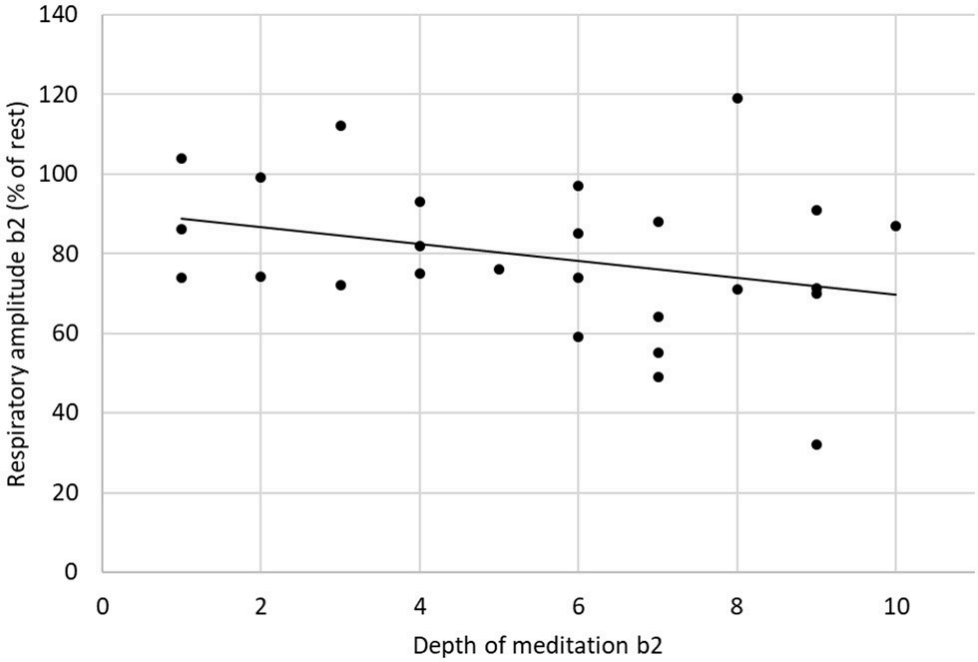
Linear correlation between respiratory amplitude and depth of meditation (ρ = −0.3297, *p* = 0.05).

The respiratory frequency was significantly higher (*p* < 10^−5^ in b1 and *p* = 0.02 in b2) during meditation when compared with rest condition (Figure 2) As illustrated on Figure 3, a significant positive linear relationship exists between the increase in the depth of meditation and the respiratory rate (ρ = 0.9685, *p* = 0.02). As shown on Figure 4, respiratory amplitude was significantly lower during meditation b1 and b2 when compared to rest condition (*p* ≤ 0.0006). Moreover, there is a weakly significant negative correlation between respiratory amplitude and the depth of meditation (Figure 5; ρ = −0.3297, *p* = 0.05).

## Discussion

The goal of our study was to investigate the effect of HM on the autonomic nervous system and the potential influence of the breathing rhythm on the HRV and rHRV. We found a reduction of some HRV and rHRV measures during HM. Unlike studies using other types of meditation, we did not found an increased parasympathetic tone during HM.

The decreases in SD_RR_, CV_RR_, and TP and the increases in LFP, nLFP, and nrLFP during deep meditation period as compared with those at rest suggested that the global vagal modulation was suppressed while the sympathetic modulations and baroreflex of the subjects were enhanced during deep medication. The increases in LFP, nLFP, and nrLFP during deep meditation period as compared with those during paced breathing suggested that deep meditation could increase both vagal and sympathetic modulations and baroreflex of the subjects. At the end of meditation, the increases in LFP, rLFP, nLFP, nrLFP, LHR, and rLHR and the decrease in rVLFP, nHFP, and nrHFP also suggested that vagal modulation was decreased while the sympathetic modulation and baroreflex were increased by deep meditation, as compared with paced breathing.

The effect of paced breathing on autonomic nervous modulation could also be observed by comparing the HRV and rHRV measures during paced breathing with those at rest. The decreases in SD_RR_, CV_RR_, TP, LFP, rLFP, nLFP, nrLFP, LHR, and rLHR and the increases in nHFP and nrHFP during paced breathing suggested that paced breathing could suppress the sympathetic modulation and baroreflex, and enhance the vagal modulation.

It is interesting to note that the effects of meditation on the HRV indices were very similar to the effects of meditation on the rHRV indices except the TP and rTP. This is comprehensible because the rTP is the residual total power after the removal of the power law function from the HRV spectrum. Despite of some studies highlighting the differences in respiratory rhythm at rest (14) linked with years of practice, we couldn't observe any correlation between experience and a reduction in respiratory rhythm at rest in our experiment.

In order to assess the changes that could be acquired by years of meditation practice, self-reported scale has been used such as in other studies (15–17). For this purpose, we used MAAS and FMI to evaluate an improvement in attention capacity and in mindfulness. But the results didn't show any correlation between scores and years of experience.

Very few studies on meditation have assessed the depth of meditation. Yet the meditative state is a subjective state that could vary from one moment to another. The Meditation Depth Questionnaire (the sole questionnaire we found in the literature) adapted from Ott (18) and Thomas and Cohen (9) was submitted to the subject to ensure that the subject was well-immersed in meditation during the data recording. We selected experimented “meditators,” who were aware of the quality of their meditation state. The Meditation Depth Questionnaire was submitted to the participants right after finishing the meditation practice. Our approach is similar to the pain scale used in a variety of medical and surgical settings to determine the severity, type, and duration of the pain. Similarly, we can quantify the meditation depth by scoring it from 1 to 10, with 10 represents the most profound state of meditation. This is a limitation of our study because we rely on the feeling of the participant. Even if this tool is subjective, it allowed us to estimate the correlation between the meditation depth and the change in the breathing rate in subjects.

These data showed a significant linear correlation between the depth of meditation and the breathing rate. In other words, deeper is the meditative state and higher is the breathing rate. This correlation has, to our knowledge, not yet been observed in other studies.

We have shown that HM did not modify the heart rate but reduce HRV significantly (SD_RR_, CV_RR_, TP, VLFP, LFP, HFP, LHR). If we look more closely at the results we have obtained, during meditation, an increase in HFnorm alone, which may reflect a modulation of the parasympathetic during meditation. But this increase is not sufficiently significant compared to the rest condition. While all the variables of HRV decrease during meditation, this increase could be explained, in part, by a very significant decrease in low frequencies (in absolute values and in normalized units). Thus, if there is a significant decrease in LF, the HFnorm can increase in an exaggerated way while the HF in the total spectrum decrease. This phenomenon has been already highlighted by Krygier et al. (17).

The use of variables in absolute values or in normalized units could also explain the disparity of the results obtained in terms of frequencies in studies to interpret an increase in parasympathetic tone during meditation. Indeed, some studies observe an increase in low frequencies (LF) interpreted as a modulation of the parasympathetic or sympathetic system or attributed to the degree of expertise of the subject or the task requested (19–21) or slow breathing rate (22). In contrast, other studies attributed the increase in HRV to an increase in normalized high frequencies (HF) together with a decrease in normalized LFs and interpreted as a predominance in vagal tone (17, 23, 24). Supplementary Table 1 summarizes the results published in literature showing the discrepancy between the studies in connection with the types of meditation.

Besides the interpretation that each author can give to these variables, we can also observe that the breathing parameters and the type of meditation could explain the heterogeneity of the results in terms of frequencies. A study conducted in 2015 trying to answer to the question “*Is meditation always relaxing?*” showed that depending on the type of meditation, frequency variations are different. They concluded that observing-thoughts meditation and loving-kindness meditation leaded to an increased stimulation of the sympathetic system compared to breathing meditation (25).

Our finding showed that both conditions, “meditation” and “paced breathing” had an effect on reducing variables such as the standard deviation of RR intervals (SD_RR_), the coefficient of variation RR (CV_RR_) and the total spectral power (TP). However, the effects of these two conditions cannot be distinguished statistically. So, it did not allow us for these variables to conclude to a meditation effect different from the respiration in terms of an overall decrease in HRV.

On the other hand, the statistically significant decrease in the low frequency frequencies (LF) (LFP, rLFP, nLFP, nrLFP) and the low-/high- frequency ratio (LHR) would be due solely to a breathing effect. The only statistically significant increase in the HRV spectrum was normalized high frequencies (HFnorm) which would also be solely due to an effect breathing.

Respiratory rhythm is often associated with psychological well-being and meditation seemed to lead to a decrease of it Wielgosz et al. (14), Nijjar et al. (23). In our study, the respiratory rhythm of subjects increased significantly during meditation compared to the rest condition. The breathing amplitude is another important data to be taken into account in this analysis. Indeed, compared to the “meditation” condition, the breathing amplitude in “paced breathing” condition is significantly higher. The subject thus has to breathe in a range and to a respiratory rate higher than the one he has at rest, so in a condition close to hyperventilation.

Posture and breathing may influence the HRV. The most determining factor in the amplification of the HRV is a slow and deep breathing rhythm (26). Rapid breathing or hyperventilation would impact on the HRV, but would induce an increase in the HFnorm, which could result from an increased tidal volume and respiration rate (27). Critchley et al. (28) examined the effects of hypoxia and slow breathing on the HRV, and assessed the neural substrates using fMRI. They noted during hypoxia a decrease in the HRV and a suppression of the baroreflex, as represented by the decrease in the LF. The authors found that dorsal medullary and pontine activity correlated positively with tidal volume and correlate inversely with heart rate. The activity in rostroventral medulla was correlated with blood pressure and HRV. We speculate that the modifications in these centers closely associated with sympathetic regulation may explain the impact of HM on sympathetic tone. A fMRI study combined with cardiovascular and respiratory measures should be performed to test this hypothesis. A study about the effects of relaxation and hyperventilation in anxious people, shows that relaxation does not really affect HFnorm and HRV. However, hyperventilation induces a decrease in HRV and an increase in HFnorm, such as our study (29).

## Conclusion

We found that HM could induce a suppression of global vagal modulation and increase the sympathetic modulation and baroreflex. In addition, imposed breathing rhythm could suppress the sympathetic modulation and enhance the vagal modulation. Unlike studies using other types of meditation, we did not identify evidence of increased vagal tone during HM. Our results suggest that the changes in breathing which occur during meditation influence HRV.

## Ethics Statement

The study was approved by the Ethical Committee of ULB Erasme (Reference 2016-521). All participating subjects signed a written informed consent after full explanation of the experimental procedures and before the experiment.

## Author Contributions

AL, SC, and MM: design of the study. AL: data collection. AL, SC, C-DK, and MM: data analysis and drafting and approval of the final version.

### Conflict of Interest Statement

The authors declare that the research was conducted in the absence of any commercial or financial relationships that could be construed as a potential conflict of interest.
