# Supplementary material for: Changes in Heart Rate Variability During Heartfulness Meditation: A Power Spectral Analysis Including the Residual Spectrum
Source: Front Cardiovasc Med. 2019 May 14;6:62. doi: 10.3389/fcvm.2019.00062 (PMC6527777; doi:10.3389/fcvm.2019.00062)
Supplement: Supplementary file 1 [file Table_1.DOCX]

**Supplementary Material**

**Table 1. Effects of Meditation on HRV and Respiratory Rate**

| Meditation effects on the HRV and the respiratory rythm | | | | | | | | | | | | | |
| --- | --- | --- | --- | --- | --- | --- | --- | --- | --- | --- | --- | --- | --- |
| Author | | Population | | Design | | Intervention | | Comparison | | HRV | | Respiration | |
| Krygier, J. R., Heathers, J. A., Shahrestani, S., Abbott, M., Gross, J. J., & Kemp, A. H. (2013). Mindfulness meditation, well-being, and heart rate variability: a preliminary investigation into the impact of intensive Vipassana meditation. *International Journal of Psychophysiology*, *89*(3), 305-313. | | 36 beginners  Enrolled in a retreat  age : +/-43 years | | Vipassana  Meditation  2 sessions:  5 min rest  5min méditation focus on breathing | | Data has been collected 1 day before retreat and +/- 4 days after retreat Between 15 and 18h | | Long-Term meditators : before and after the retreat  Analysing HRV-Well Being | | Meditation Vs Rest :  ↑Absolute HF power (ln HF)  HF n.u no significant  LF no significant  Interaction time x meditation task (1X2)  ↑nu HF  ↓THM (=LF) during meditation after retreat. | |  | |
| [Takahashi](http://www.citeulike.org/user/dr0x29a/author/Takahashi:T) T.,  [Murata](http://www.citeulike.org/user/dr0x29a/author/Murata:T) T.,  [Hamada](http://www.citeulike.org/user/dr0x29a/author/Hamada:T) T.,  [Omori](http://www.citeulike.org/user/dr0x29a/author/Omori:M) M., [Kosaka](http://www.citeulike.org/user/dr0x29a/author/Kosaka:H) H.,  [Kikuchi](http://www.citeulike.org/user/dr0x29a/author/Kikuchi:M) M., [Yoshida](http://www.citeulike.org/user/dr0x29a/author/Yoshida:H) H.,  [Wada](http://www.citeulike.org/user/dr0x29a/author/Wada:Y) Y., (2005) Changes in EEG and autonomic nervous activity during meditation and their association with personality traits**,** *International Journal of Psychophysiology*, Vol. 55, No. 2., pp. 199-207. | | n=20  male student  +/- 25 years  beginners | | Zen meditation Laying down  Control of breathing and attention  5 min of stabilization  15 min. rest  30 min. break | | EEG, RR, ECG ( HRV)  Counting from 1 to 100 focusing on breathing with a metronome.0. 25Hz  EEG and ECG + breathing | | EEG HRV  character traits  Rest Vs méditation | | ↑ nuHF meditation  ↓nuLF et LF/HF ratio | |  | |
| Tang, Y. Y., Ma, Y., Fan, Y., Feng, H., Wang, J., Feng, S., ... & Zhang, Y. (2009). Central and autonomic nervous system interaction is altered by short-term meditation. *Proceedings of the national Academy of Sciences*, *106*(22), 8865-8870. | | n=80 students beginners  4 groups:  -IRM+Physio  n=20 meditation  n=20  control group  -EEG+ Physio  n=20 meditation  n=20 control group | | Méditation Mindfulness  Formation 5 jours/ 20 min./j | | 5 periods for data collection: baseline data before training, 3 x 9 min training period and baseline data after treatment | | Meditation IBMT Vs control group | | ↑(nuHF) HRV meditation group VS relaxation group.  ↓RC in a significative way | | ↑ abdominal respiratory amplitude and ↓ Thoracic RR in the meditation group Vs relaxation | |
| Peng, C. K., Henry, I. C., Mietus, J. E., Hausdorff, J. M., Khalsa, G., Benson, H., & Goldberger, A. L. (2004). Heart rate dynamics during three forms of meditation. *International journal of cardiology*, *95*(1), 19-27. | | 11 Kundalini yoga experienced, 3 to 5 years, 5 times a week. | | 3 interventions in a session:  ECG (chest)  Abdominal and thoracic straps  10 min of each practice  Preceded by a rest of 10 min (control) | | Meditation: focus on a mantra  Fire breathing: fast breathing: 140 / min, thoracic level  Segmented breathing: inspiration and expiration on 8 beats | | Comparing rest VS intervention | | **Meditation :**  RC : ns  ↑HRV ↑LF  **Fire Breathing** ↑RC  ↓VRC ↓TP  ↓LF ↑HF  **Slow breathing:**  ↑RC:  ↑HRV  ↑LF  ↓HF/LF Ratio | |  | |
| Delgado-Pastor, L. C., Perakakis, P., Subramanya, P., Telles, S., & Vila, J. (2013). Mindfulness (Vipassana) meditation: Effects on P3b event-related potential and heart rate variability. *International Journal of Psychophysiology*, *90*(2), 207-214. | | n=10 experienced | | Vipassana  MEditation  2sessions :  -5-minute rest -Listening an audio  - 30-minute meditation/wandering thoughts  -listening an audio | | ECG et EEG | |  | | Méditation  ↑HRV  ↑LF | | Not taken into account | |
| Wu, S. D., & Lo, P. C. (2008). Inward-attention meditation increases parasympathetic activity: a study based on heart rate variability. *Biomedical Research-Tokyo*, *29*(5), 245-250. | | n= 10 experienced  n= 10 control group | | Zen Meditation  One session with 2 interventions :  Rest and meditation | | The meditator focuses his attention on a Zen chakra at the level of the heart ECG and waist belt at the nave | | Compare HRV  control group Vs experienced | | Meditation : No effect on RMSSD  ↑TP  ↓LF  ↓LF/HF ratio  ↑HF | | Respiration rythm : no significant changes | |
| Phongsuphap, S., Pongsupap, Y., Chandanamattha, P., & Lursinsap, C. (2008). Changes in heart rate variability during concentration meditation. *International journal of cardiology*, *130*(3), 481-484. | | n=35 experienced  (teacher of meditation)  n=17  volunteers  control group  SITTING position !!! | | 4 weeks.  5 periods for data collection: baseline data before training, 3 x 9 min training period and baseline data after treatment | | 5 min at rest  10 min meditation focused on breathing | | Effects on HRV Domain temp. : DRQL RC Frequency domain: nu HF, nu LF, nuVLF | | During medtation :  ↑ HRV  ↑nuLF | | Not taken into account | |
| Peressutti, C., Martín-González, J. M., García-Manso, J. M., & Mesa, D. (2010). Heart rate dynamics in different levels of Zen meditation. *International journal of cardiology*, *145*(1), 142-146. | | n=19 experienced  G1: n=5:  4.5 years-experienced  G2: n=4:  16 years-experienced  G3 :13,  6 years +/- 5 years | | Zen Soto meditation 2-4 sessions on a one-month retreat with rest and meditation.  -10 min rest  -40 min of meditation  -20 min AF: Focusing on breathing  20 min MM: Without directed attention, observing what happens. | | Sitting position,  ECG and RR recording | | Comparison of 3 groups  Rest data Meditation and breathing in frequency domain | | ↑HRV  LF predominate especially in  G3. HF ↓  MM Mindfulness  G1 LF-HF  FA Focused Attention  G1 :VLF  MM  G3 (11-20 years experienced)  FA  G3 ↑LF ++ | | The less experience there is and the higher the RR is. With the experienced subjects, the RR oscillate more. | |
| Wielgosz, J., Schuyler, B. S., Lutz, A., & Davidson, R. J. (2016). Long-term mindfulness training is associated with reliable differences in resting respiration rate. *Scientific reports*, *6*, 27533. | | Méditation Mindfulness  n =31 experienced  minimum 3 years with a daily practice of 30 min. + have participated in at least 3 retreats. n = 34 Control Group | | 3 lab sessions spaced by 4.5 months .  24h-Lab session The 2 and 3 sessions were preceded by a session of 8 hours of training the day before.  6 min recording at rest with plethysmography. Time domain analysis | | Collecting RR data at rest  Questionnaire, demographic, physiological, behavioral and neuroimage data | | Relation between respiratory rhythm at rest and years of experience in meditation  Compare meditating and non-meditating  Compare effect of intensive retreat and daily practice of short duration | | _________________ | | Inverse Relation:  1- year-experience corresponds to a decrease of 0,16 bpm ↓ RR  RR rest of experienced beginnersVs:   -1.6 cpm  + Effect of an intensive retreat Vs daily practice  Double hour of retreat  with a decrease of 0, 7 bpm | |
| Studies showing very few effects on HRV | | | | | | | | | | | | | |
| Chu, I. H., Wu, W. L., Lin, I. M., Chang, Y. K., Lin, Y. J., & Yang, P. C. (2017) Effects of Yoga on Heart Rate Variability and Depressive Symptoms in Women: A Randomized Controlled Trial. *The Journal of Alternative and Complementary Medicine*. | | n = 26 depressive women beginners  -n=13 trainned in yoga -n=13 control group | | 2 sessions: ECG recording of 20 minutes  Laying down! | | 8 weeks of training 2 times a week, 60 min.  5 min breathing exercises  5 min warm up  40 min posture  10 min meditation | | Depression and HRV Before and After Yoga Training | | -RC does not change -SDNN not significant  ↑ HF  ↓ LF  ↓ LF / HF ratio | | Not taken into account | |
| Lumma, A. L., Kok, B. E., & Singer, T. (2015). Is meditation always relaxing? Investigating heart rate, heart rate variability, experienced effort and likeability during training of three types of meditation. *International Journal of Psychophysiology*, *97*(1), 38-45. | | n = 160 beginners  n = 80 started with a module n = 80 started with another module Each module over 3 months | | Longitudinal study (9 months) Training in a type of meditation for 3 months for each group HF-HRV analysis as parasympathetic and RC as sympathetic | | Collecting data at week 3 and 13 of each module. During each module, training minimum 5 times / week with a 20 minute audio guide. + 2h weekly session | | Compare RC and HF-HRV and level of effort during 3 types of meditation: breathing, compassion, open to thoughts | | Lower RC during breathing meditation than compassion or open to thoughts. RC ↑ and ↓ HF-HRV decreases with training for these same 2 meditations | | Not taken into account. | |
| *Steinhubl SR, Wineinger NE, Patel S, Boeldt DL, Mackellar G, Porter V, Redmond JT, Muse ED, Nicholson L, Chopra D and Topol EJ (2015) Cardiovascular and nervous system changes during meditation. Front. Hum. Neurosci. 9:145.* | | n=20 experienced :  regular practice for 3 months: + - 32 min / day n = 20beginners | | 2 session: 1 day of retreat and after retirement (7 days) meditation mantra ECG, EEG, HR, RR, HRV | | . 26 min silent mantra meditation  . 20 min listening talks  .4 min: Guided Breathing Exercises | | observe individual variations via continuous EEG monitoring, blood pressure, CR and HRV in novices and experienced | | RC: No change  ↓ RMSSD experienced  -Meditation: no change HRV  -Slow breathing ↓ nu HF (experienced and novices | | RR no significant change although a little slower in both groups during meditation. | |
| Nijjar, P. S., Puppala, V. K., Dickinson, O., Duval, S., Duprez, D., Kreitzer, M. J., & Benditt, D. G. (2014). Modulation of the autonomic nervous system assessed through heart rate variability by a mindfulness based stress reduction program. *International Journal of Cardiology* *177*(2), 557-559. | | n= 18 beginners | | Mindfulness Meditation Class of 2-3h weekly during 8 weeks + daily practice. | | -5 min. rest  -5 min. breathing 6cycles per minute  -5 min. meditation, sitting with K7 audio | | Compare the change before and after the Mindfulness program based stress program | | No change in RC  -TP, HFP, LFP no change  -After 8 weeks and during meditation  ↓ Nulf  ↑ HFnu  -Slow breathing  ↑nuLF ↓nuHF, LFP,↑ | | ↓ Respiration  during meditation versus rest | |
| Kim, D., Kang, S. W., Lee, K. M., Kim, J., & Whang, M. C. (2013). Dynamic correlations between heart and brain rhythm during Autogenic meditation. *Frontiers in human neuroscience*, *7*, 414.   \|  \|  \| \| --- \| --- \| | | n=12 beginners  of a local autogenic meditation community | | Autogenic meditation  EEG, ECG  Sitting, Closed eyes  .5 min sitting rest.  .5 to 15 minutes of meditation | | Training of 8 weeks followed by a month of daily meditation. Photoplethsmography  EEG | | EEG and ECG change before and after | | Global HRV has not changed. HF did not change compared to rest. Cardiac coherence changes rather than LF | |  | |
| Pittig, A., Arch, J. J., Lam, C. W., & Craske, M. G. (2013). Heart rate and heart rate variability in panic, social anxiety, obsessive–compulsive, and generalized anxiety disorders at baseline and in response to relaxation and hyperventilation. *International journal of psychophysiology*, *87*(1). | | 89 subjects with primary anxiety disorders according to DSM-IV 39 control subjects | | One session with 2 interventions Relaxation and Hyperventilation 30 min | | -5 min at rest  -15 min relaxation -1 min in hyperventilation 76cpm CO2 taken during hyperventilation to lower them by 50% CO | | Compare the HF-HRV At rest Relaxation hyperventilation Compare the HF-HRV between the control group and people with anxiety | | HF-HRV does not change significantly between rest and relaxation. ↑ HF-HRV during hyperventilation | |  | |
| Oxygen consumption during medtitation | | | | | | | | | | | | | |
| Tyagi, A., Cohen, M., Reece, J., & Telles, S. (2014). An explorative study of metabolic responses to mental stress and yoga practices in yoga practitioners, non-yoga practitioners and individuals with metabolic syndrome. *BMC complementary and alternative medicine*, *14*(1), 445. | .YP (n = 16) Practicing yoga for at least 6 months, 90 min / day  . NY (n = 15)  . MS (n = 15) Patient with metabolic problem | | | 1 session with 9 sequences:  4 interventions (5min.) interspersed with rest period | | -Induction of stress by arrhythmic exercices  -Rest  Respiration by a nostril -Rest  -Fast Breathing: 48rpm  -Rest  -Meditation (mantra)  Laying down | | Effect of yoga on recovery from stress and on O2 consumption during breathing or meditation Comparison of the three groups. | | ______________ | | YP have a lower O2 consumption at rest compared to the other 2 groups.  YP has greater variability in O2 consumption in different equations.  YP has a better recovery compared to induced stress | |
| Attentional tasks reduce the HRV | | | | | | | | | | | | | |
| Keller, J., Bless, H., Blomann, F., & Kleinböhl, D. (2011). Physiological aspects of flow experiences: Skills-demand-compatibility effects on heart rate variability and salivary cortisol. *Journal of Experimental Social Psychology*, *47*(4), 849-852. | 8 students from University | | | HRV assessment and other questionnaire measures One session with 3 different conditions:  3 min at rest  5min condition1  5 min condition2  5 min Condition 3 | | A task of knowledge is requested, answer to questions of general knowledge.  3 conditions:  -Boredom  -under level adapted at S level  - Above the level of S | | Comparison of HRV behavior during the 3 conditions | | More task involves attention and commitment and more HRV ↓ | | Not taken into account | |
| Effects of slow breathing or HRV biofeedback | | | | | | | | | | | | |  |
| Author | | | Population | | Study Design | | Intervention | | Comparison | | HRV | |  |
| Giardino, N. D., Chan, L., & Borson, S. (2004). Combined heart rate variability and pulse oximetry biofeedback for chronic obstructive pulmonary disease: preliminary findings. *Applied psychophysiology and biofeedback*, *29*(2), 121-133. | | | n=20 COPD (Gold II) | | 9 training sessions: -5 weekly sessions.  HRV-biofeedback seated - 4 weekly sessions. : walking + Biofeedback Saturometer (+ Respi) | | HRV Biofeedback: ECG electrodes, saturometer, velcro strap abdo. Walk : With saturometer and encouragement respi biofeedback | | Comparison before / after  -Test of 6 min walk.  -Questionnaire of Saint-Gilles Respiratory on quality of life | | ↑ HRV  ↑ LF  -Test of 6 min walk: Significant improvement -Questionnaire Saint-Gilles ↑ -Dyspnea ↓ -R spontaneous R at rest | |  |
| Paul, M., & Garg, K. (2012). The effect of heart rate variability biofeedback on performance psychology of basketball players. *Applied Psychophysiology and Biofeedback*, *37*(2), 131-144. | | | n = 30 basketball players  n = 10 HRV / B  n = 10 placebo group  n = 10 control group | | 10 consecutive days HRVB training. questionnaires | | 3min dribble, pass and shoot. Then plethysmographic evaluation with digital sensor and abdominal belt for HRV and RR measurements | | Compare before after and one group to another: anxiety and performance of athletes | | HRVB Group  ↑ HRV  ↑ LF  ↓ HF  Increased performance in the 3 physical tests. | |  |
| Tsai, H. J., Kuo, T. B., Lee, G. S., & Yang, C. C. (2015). Efficacy of paced breathing for insomnia: enhances vagal activity and improves sleep quality.  *Psychophysiology*, *52*(3), 388-396. | | | n = 14 subjects with insomnia Pittsburgh Sleep Quality Index (PSQI) questionnaire n = 14 good sleep subjects | | 2 sessions spaced a week | | 5 min at rest 20 min of breathing imposed at (0.2Hz = 12 cpm) / (0.1Hz = 6cpm) 5 min rest just before sleeping.  One week at 0.2Hz and the other week at 0.1Hz | | Compare insomniac group and control group: HRV and quality of sleep. Evaluate the effect of slow breathing on sleep quality and HRV | | During slow breathing 0.1Hz (6cpm) at insomniac VS normal breathing and + significant Vs group control. ↑ HRV  ↑ TP  ↓ nuHF  ↑ nuLF | |  |
| Howorka, K., Pumprla, J., Tamm, J., Schabmann, A., Klomfar, S., Kostineak, E., ... & Sovova, E. (2013). Effects of guided breathing on blood pressure and heart rate variability in hypertensive diabetic patients. *Autonomic Neuroscience*, *179*(1), 131-137. | | | n = 32 n = 16 treatment subjects n = 16 control group | | 8 weeks slow breathing training (<10cpm) 15 min / day | | HRV recording sequence of 15 min in different positions (lying, sitting, standing).  24-hour BP recording | | Medium-term effects of slow breathing device on PS and HRV in hypertensive diabetic patients | | HRV ↑  SDRR no change  ↑ TP  ↓ nuHF  ↑ nuLF  ↓ BP | |  |
| Sowder, E., Gevirtz, R., Shapiro, W., & Ebert, C. (2010). Restoration of vagal tone: a possible mechanism for functional abdominal pain. *Applied psychophysiology and biofeedback*, *35*(3), 199-206. | | | n = 20 children with abdominal pain n = 10 children with pain control group | | Mobile acquisition unit to wear 4h / day for 8 weeks. 6 sessions HRVBiofeedback + 10 min slow breathing / day | | 5 min. recording several times a day for 8 weeks | | Compare before-after treatment: HRV and intensity and frequency of symptoms | | For the group with pain and HRVBiofeedback treatment,  ↑ nuLF during treatment. Decreased intensity and frequency of pain with better regulation of vagal tone after 8 weeks | |  |
| Hallman, D. M., Olsson, E. M., Von Schéele, B., Melin, L., & Lyskov, E. (2011). Effects of heart rate variability biofeedback in subjects with stress-related chronic neck pain: a pilot study. *Applied psychophysiology and biofeedback*, *36*(2), 71-80. | | | n = 21 subjects with neck pain and stress  n = 11 treatment  n = 10 control (just breathing) | | 10 weeks of weekly HRVB sessions + slow breathing (15min / day) | | HRV Recording  -10 min at rest  Stress tests:  -Hand stress grip  -Cold Pressure test -Deep breathing test | | Before and after protocol | | For the treatment group:  HRV at rest  ↑ HRV  ↑ LF  -During the Hand grip test:  ↑ SDRR  -Cold Pressure test:  ↑ HRV better test response | |  |
